# Neural Tube Defects and Folate Pathway Genes: Family-Based Association Tests of Gene–Gene and Gene–Environment Interactions

**DOI:** 10.1289/ehp.9166

**Published:** 2006-06-15

**Authors:** Abee L. Boyles, Ashley V. Billups, Kristen L. Deak, Deborah G. Siegel, Lorraine Mehltretter, Susan H. Slifer, Alexander G. Bassuk, John A. Kessler, Michael C. Reed, H. Frederik Nijhout, Timothy M. George, David S. Enterline, John R. Gilbert, Marcy C. Speer, the NTD Collaborative Group

**Affiliations:** 1 Center for Human Genetics, Duke University Medical Center, Durham, North Carolina, USA; 2 Feinberg School of Medicine, Northwestern University, Chicago, Illinois, USA; 3 Department of Mathematics and; 4 Department of Biology, Duke University, Durham, North Carolina, USA; 5 Department of Surgery and; 6 Department of Radiology, Duke University Medical Center, Durham, North Carolina, USA

**Keywords:** folate, folic acid supplementation, genetic association, neural tube defects

## Abstract

**Background:**

Folate metabolism pathway genes have been examined for association with neural tube defects (NTDs) because folic acid supplementation reduces the risk of this debilitating birth defect. Most studies addressed these genes individually, often with different populations providing conflicting results.

**Objectives:**

Our study evaluates several folate pathway genes for association with human NTDs, incorporating an environmental cofactor: maternal folate supplementation.

**Methods:**

In 304 Caucasian American NTD families with myelomeningocele or anencephaly, we examined 28 polymorphisms in 11 genes: folate receptor 1, folate receptor 2, solute carrier family 19 member 1, transcobalamin II, methylenetetrahydrofolate dehydrogenase 1, serine hydroxymethyl-transferase 1, 5,10-methylenetetrahydrofolate reductase (*MTHFR*), 5-methyltetrahydrofolate-homo-cysteine methyltransferase, 5-methyltetrahydrofolate-homocysteine methyltransferase reductase, betaine-homocysteine methyltransferase (*BHMT*), and cystathionine-beta-synthase.

**Results:**

Only single nucleotide polymorphisms (SNPs) in *BHMT* were significantly associated in the overall data set; this significance was strongest when mothers took folate-containing nutritional supplements before conception. The *BHMT* SNP rs3733890 was more significant when the data were stratified by preferential transmission of the *MTHFR* rs1801133 thermolabile T allele from parent to offspring. Other SNPs in folate pathway genes were marginally significant in some analyses when stratified by maternal supplementation, *MTHFR*, or *BHMT* allele transmission.

**Conclusions:**

*BHMT* rs3733890 is significantly associated in our data set, whereas *MTHFR* rs1801133 is not a major risk factor. Further investigation of folate and methionine cycle genes will require extensive SNP genotyping and/or resequencing to identify novel variants, inclusion of environmental factors, and investigation of gene–gene interactions in large data sets.

Of 1,000 births worldwide, in one embryo the neural tube will fail to close properly 28 days after conception, resulting in some form of neural tube defect (NTD). Failed closure at the cranial end, known as anencephaly, is a lethal condition, whereas failed closure at the caudal end usually results in a myelomeningocele. NTDs are the most common debilitating birth defect. Familial studies indicate a significant genetic component to NTDs, with a 40-fold increase in risk in first-degree relatives (Elwood et al. 1992). Myriad environmental exposures have been implicated in the development of NTDs; most notably, a significant decrease in risk can be achieved by maternal folic acid supplementation before conception.

The mechanism by which dietary folate supplementation prevents NTDs is poorly understood (MRC Vitamin Study Research Group 1991). Folic acid derivatives are essential for the synthesis of DNA, cell division, tissue growth, and DNA methylation (Morrison et al. 1998). Methylation enables proper gene expression and chromosome structure maintenance, both of which are critical in the developing embryo (Razin and Kantor 2005). The folate and methionine cycles are linked by the conversion of homocysteine to methionine (Figure 1). In the absence of food frequency data, maternal vitamin supplementation can also serve as a proxy for overall health because of the positive correlation between supplement intake, diet, and a healthy lifestyle (Slesinski et al. 1996). Vitamin supplementation is an important cofactor to consider when studying nutritionally related genes.

Animal models demonstrate that periconceptional folate supplementation protects against congenital defects in the face, neural tube, and conotruncal region of the heart. Low folate could directly limit its availability to cells or indirectly disrupt methionine metabolism, thereby increasing homocysteine in the maternal serum (Rosenquist and Finnell 2001). Either mechanism implicates folate receptor and methionine–homocysteine regulatory genes.

Folate enters cells by folate receptor 1 [*FOLR1*; GenBank accession no. NM_016725 (http://www.ncbi.nih.gov/GenBank)] and folate receptor 2 (*FOLR2*; GenBank accession no. NM_000803) or carrier-mediated internalization by solute carrier family 19 member 1(*SLC19A1*; GenBank accession no. U15939), also known as reduced folate carrier protein 1. Transcobalamin II (*TCN2*; GenBank accession no. NM_000355) imports vitamin B_12_, cobalamin, a cofactor for another folate enzyme, 5-methyltetrahydrofolate-homocys-teine methyltransferase (*MTR*; GenBank accession no. NM_000254).The reactions within the folate metabolism cycle can be very complex, with methylenetetrahydrofolate dehydrogenase 1 (*MTHFD1*; GenBank accession no. J04031), serine hydroxymethyl-tranferase 1 (*SHMT1*; GenBank accession no. NM_004169), and 5,10-methylenetetrahy-drofolate reductase (*MTHFR* ; GenBank accession no. NM_005957) being widely studied in the NTD literature.

*MTHFR* rs1801133 is the most frequently investigated polymorphism in NTDs with conflicting results in different populations: Dutch and Irish populations associate the TT allele with risk (Shields et al. 1999; van der Put et al. 1995), whereas a protective effect is seen in Italians (De Marco et al. 2002) and other populations have no evidence of association (Gonzalez-Herrera et al. 2002; Revilla et al. 2003; Stegmann et al. 1999). This polymorphism also has a confirmed role heart disease (Frosst et al. 1995).

Homocysteine can accumulate from low dietary folate, cobalamin, and/or genetic factors (Morrison et al. 1998; Ramsbottom et al. 1997) and is elevated in some NTD mothers (Mills et al. 1995; Steegers-Theunissen et al. 1994). Homocysteine itself may be teratogenic (Rosenquist et al. 1996) or impair substrates for methylation reactions (Essien and Wannberg 1993). Enzymes that degrade homocysteine regulate homocysteine levels; for example, MTR converts homocysteine to methionine and folate to tetrahydrofolate (Trembath et al. 1999). 5-Methyltetrahydrofolate-homocysteine methyltransferase reductase (*MTRR*; GenBank accession no. AF025794) maintains MTR in its active state. Betaine-homocysteine methyltransferase (*BHMT*; GenBank accession no. BC012616) remethylates homocysteine to methionine with a betaine cofactor (Morin et al. 2003). Cystathionine-betasynthase (*CBS*; GenBank accession no. NM_000071) controls homocysteine levels by degrading homocysteine into cystathionine (Morrison et al. 1998).

Detecting moderate effects of multiple folate genes will be particularly difficult if they are interactive or additive with environmental impacts (Morrison et al. 1998). This complex pathway has several known metabolic interactions, such as MTRR maintaining MTR in an active state. Previous studies found an association of *MTHFR* and *MTRR* (Gueant-Rodriguez et al. 2003; Wilson et al. 1999) plus *CBS* and the *MTHFR* thermolabile variant with NTDs (Afman et al. 2003; Ramsbottom et al. 1997; Speer et al. 1999).

Thus, genes involved in folate metabolism are compelling candidates for NTDs, from both a genetic and an environmental perspective.

## Material and Methods

### Sample population

All polymorphisms were genotyped in 304 families with at least one individual affected with an NTD and their first-degree relatives when available. These families represent 240 complete trios and 64 families with only one parent, whereas 16 of these families had two or more affected individuals. Cases with lumbosacral myelomeningocele were classified as affected in the narrow diagnostic criteria, and any level NTD was affected in the broad criteria. These Caucasian families were collected from 13 sites across the United States through myelodysplasia clinics, neuro-surgical referrals, our study website, and word of mouth. The family-based study design is robust to potential population stratification and particularly useful when sampling over such a wide geographic area. Most affected individuals were ascertained as children (average age at sample, 14.3 years) with no sex differences. In 74% of NTD case mothers, extensive environmental exposure interviews were conducted, including pre- and post-conceptional vitamin use. Table 1 outlines the sample sizes subdivided by diagnostic criteria and maternal folate supplementation. This study was approved by the Duke University Medical Center Institutional Review Board, and all data and samples were collected after informed consent of subjects.

### SNP genotyping

**Figure 2:**
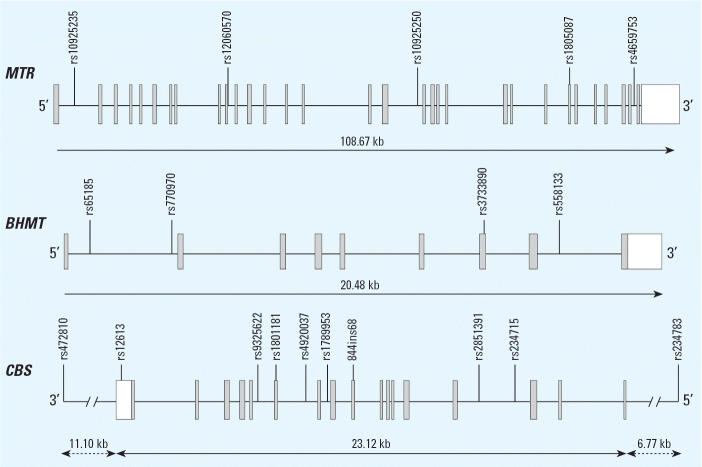
Genomic location of genotyped SNPs in relation to the three genes with three or more genotyped SNPs: *MTR*, *BHMT*, and *CBS*.

Eleven genes of the folate pathway are included in our study and were selected from previously published NTD research (Table 2). Three genes that degrade homocysteine (*MTR*, *BHMT*, and *CBS*) were more thoroughly genotyped based on HapMap Release 19 (International HapMap Project 2005) tagging single nucleotide polymorphisms (SNPs) and location in the gene (Figure 2). All but two genetic variants were genotyped by commercially available TaqMan allelic discrimination assays (Assay-on-Demand and Assay-by-Design, Applied Biosystems, Foster City, CA). Previously published poly-merase chain reaction (PCR) primers for a 68-bp insertion in *CBS* exon 8 (Morrison et al. 1998) produced results that did not pass the quality control measures outlined below. Sequencing of the insertion showed a tandem duplication such that the forward primer hybridized before and within the insertion. We used a forward primer 58 bp further upstream of the insertion producing 242 or 310 bases fragments (forward, 5′-CGGCGGTATTG-GCCACTC-3′; reverse, 5′ GGCCGGGC-TCTGGACTC-3′). The *SLC19A1* SNP rs1051266 was genotyped by melting curve analysis in the MGB Eclipse Probe System (Belousov et al. 2004). All PCR amplification used the GeneAmp PCR system 9700 thermo-cyclers (Applied Biosystems) according to assay specifications. Fluorescence was detected with the ABI Prism 7900HT Sequence Detection System and analyzed with ABI Prism Sequence Detection System software (version 2.0; Applied Biosystems). Quality control measures consisted of two reference samples from the Centre d’Etude du Polymorphisme Humain in Paris, France, and 24 duplicated samples per 384-well plate plus blinded from laboratory technicians. These 26 samples had to match completely, and at least 90% of all samples had to be successfully genotyped for the polymorphism to pass quality control. Genotypes were also checked for Mendelian inconsistencies within families.

### Statistical analysis

Family-based association analysis was performed using the pedigree disequilibrium test (PDT) (Martin et al. 2000) and association in the presence of linkage (APL) test (Martin et al. 2003). Because of the mixed family types and incomplete sampling in our data set, PDT will take advantage of multiplex families, whereas APL performs better with missing data. These tests were performed on all SNPs for the narrow and broad phenotypes in the overall data set as well as those subdivided by maternal folate supplementation, *BHMT* allele transmission, and *MTHFR* allele transmission. All SNPs were checked for Hardy-Weinberg equilibrium (HWE) separately in unrelated affected individuals and unaffected relatives in the complete data set using genetic data analysis (Weir 1996). The reported *p*-values have not been corrected for multiple testing, but a strict correction is not critical given the biological plausibility implicating these genes in NTDs. Linkage disequilibrium (LD) between the SNPs in the same gene was calculated using the Graphical Overview of Linkage Disequilibrium (GOLD) software package (Abecasis and Cookson 2000).

## Results

### Single gene associations with an environmental stratification

The initial analysis of the entire data set for 28 SNPs in 11 genes (Table 3) found associations: *BHMT* rs3733890 (narrow PDT *p* = 0.023, narrow APL *p* = 0.058, broad PDT *p* = 0.025, broad APL *p* = 0.035) and *BHMT* rs558133 (broad PDT *p* = 0.025, broad APL *p* = 0.061). All SNPs were in HWE except the *MTHFD1* SNP rs2236225 in affected individuals only (data not shown). When subdivided by case mothers’ dietary supplementation with folate 3 months before conception, the *BHMT* associations were significant only in the supplemented group: rs3733890 (narrow PDT *p* = 0.027, narrow APL *p* = 0.055, broad PDT *p* = 0.016, broad APL *p* = 0.027) and rs558133 (narrow PDT *p* = 0.036, broad PDT *p* = 0.012).

When all SNPs were analyzed in the stratified data set, two other genes had significant associations (Table 3). *MTHFR* rs1801133 was associated by APL with the narrow phenotype in families that did not supplement (*p* = 0.046). Also in the nonsupplementing families, *CBS* was associated by PDT with the broad phenotype in rs234715 (*p* = 0.015) and rs4920037 (*p* = 0.037) and SNPs in *MTR* showed significance: rs1092535 (narrow PDT *p* = 0.066, narrow APL *p* = 0.031, broad PDT *p* = 0.040, broad APL *p* = 0.04) and rs4659743 (narrow APL *p* = 0.013, broad PDT *p* = 0.041, broad APL *p* = 0.010). Despite being 96.6 kb apart, high LD (*D*′ = 0.973, *r*^2^ = 0.946) throughout *MTR* could account for both SNPs’ associations (Table 4).

### Stratifying by other genes

In complex conditions like NTDs, multiple genes are likely contributing to folate-related risk. To evaluate multigenic effects, families were grouped by preferential transmission of an allele to affected offspring and reevaluated for all other SNPs. For *BHMT* rs373389, 79 families preferentially transmitted the G allele, 59 transmitted the A allele, 149 transmitted both equally or had homozygous parents, whereas 17 could not be determined and were not included in the analysis (Table 5). When the G allele was preferentially transmitted, the *CBS* insertion was significant by PDT (*p* = 0.033 for both diagnostic groups), whereas two SNPs were significant by APL: *SHMT* rs1979277 (*p* = 0.042 narrow, *p* = 0.020 broad) and *MTR* rs4659743 (*p* = 0.049 narrow, *p* = 0.015 broad). When segregating the A allele, *MTHFD1* rs2236225 was significant by PDT in the broad phenotypic group (*p* = 0.016). Other SNPs in *BHMT* were significant in the stratified groups due to inter-marker LD (Table 4).

We performed a similar analysis stratifying by transmission of the *MTHFR* rs1801133 thermolabile T allele (Table 6). Sixty-eight families were grouped for the T allele; 90 families were grouped for the C allele; 134 families did not preferentially transmit either allele; and 12 were excluded. With overtransmission of the T allele, *BHMT* rs3733890 is more significant than in any prior analysis (narrow PDT *p* = 0.007, narrow APL *p* = 0.027, broad PDT *p* = 0.010, broad APL *p* = 0.047), and *TCN2* rs1801198 was associated by PDT with the broad phenotype (*p* = 0.045). For the C allele subset, rs1801394 in *MTRR* was significant by APL in the broad group (*p* = 0.048). When neither allele was preferred, the *SHMT* SNP is significant by PDT (*p* = 0.050 for narrow, 0.037 for broad).

## Discussion

### BHMT contributes to the risk of NTDs

*BHMT* is significantly associated with NTDs in our sample set, particularly when mothers were receiving preconceptional folate or parents preferentially transmitted the *MTHFR* rs1801133 T allele. It is not immediately apparent how *BHMT* would increase NTD risk in a folate-rich environment. In adults, BHMT functions predominantly in the liver, whereas MTR is active in all tissues (Zhu et al. 2005), but the expression patterns in the developing embryo are unknown and may be markedly different than that in the adult. BHMT is responsible for up to 50% of methylation in the adult liver (Finkelstein and Martin 1984).

The methyl cycle supplies 1-carbon units critical for a variety of methylation reactions essential for proper gene expression and maternal and paternal imprinting by methylated DNA (Razin and Kantor 2005). Growth factor genes are commonly imprinted in this manner, and nutrition can alter these methylation patterns (Waterland and Jirtle 2003). Faulty embryonic methylation of DNA due to abnormal folate levels or improper methyl cycle gene expression at a critical developmental juncture could inappropriately silence growth factors necessary for proper tube closure.

Homocysteine levels are also maintained by the methyl cycle and play a role in NTD risk. Large-dose oral betaine therapy, a BHMT cofactor, treats hyperhomocysteinemia by shunting homocysteine through a betaine-dependent remethylation pathway (Kang 1996). When folate dependent methio-nine synthesis is impaired, by either genetic or environmental factors, BHMT plays a critical role in homocysteine homeostasis (Weisberg et al. 2003). However, the *BHMT* R (G allele) and Q (A allele) proteins show no differences in thermostability or enzymatic Michaelis constant (Q = 2.7 and R = 2.8) (Weisberg et al. 2003). The association of hyperhomocysteinemia with NTD risk implicates enzymes such as MTR, BHMT, and CBS that degrade homocysteine.

Our observed relationship between *BHMT*, folate supplementation, and NTD risk appears counterintuitive. It is possible that the stratification method inadvertently grouped families by an unidentified cofactor correlated with supplementation. The *BHMT* polymorphism could also create a highly efficient variant that causes the metabolic cycles to overfunction when combined with high folate levels. Human NTDs can only be studied at birth, not at the true point of incidence 28 days postconception, so we may fail to observe a high-risk group incompatible with life. Such individuals with insufficient BHMT and low folate may not be observable unless they also have an additional unknown protective factor. All these hypotheses are highly speculative, particularly in the absence of any biological support.

In the subset of families also transmitting the *MTHFR* T allele, affected children who have inherited at least one copy of the thermolabile allele from a heterozygous parent are even more likely to have also received the *BHMT* A allele. A gene–gene interaction between *MTHFR* and *BHMT* would require polymorphisms in both genes for the disorder, or additional correlated factors are involved and undetectable in this sample. These results implicate *BHMT* in NTD risk alone, in conjunction with maternal folate supplementation, and/or a polymorphism in *MTHFR* that proper folate metabolism.

### Other folate pathway genes implicated

The most widely studied gene in NTD research, *MTHFR*, is not a significant risk factor in our overall data set. In families that did not receive folate supplementation, the rs1801133 polymorphism was moderately significant. Significant prior research combined *MTHFR* with other genes, and our results found *BHMT* to be highly significant in the T allele subgroup.

*MTHFR* rs1801133 is not the only genetic NTD risk factor, particularly in Caucasian Americans. Some NTD cases are not folic acid preventable, and at most 25% of cases can be solely explained by rs1801133 (Posey et al. 1996; van der Put et al. 1996). Excluding TT genotype people, there is still a decrease in folate and increase in homocysteine levels in patients and their parents (van der Put et al. 1997).

Some previously investigated NTD-related genes included in this study are less likely to be involved because of their biochemical function. For example, FOLR2 is not the primary binder of folate, therefore the lack of significant association does not contradict models of folate metabolism (Trembath et al. 1999). Mathematical models of the folate and methionine cycles indicate that these systems are quite robust to dietary folate intake and perform well without significant folate intake for several months (Nijhout et al. 2004).

Conversely, lack of significance does not rule out their involvement in the etiology of NTDs. Under a dominant model with a baseline risk of 0.0001 and a genetic relative risk of 0.6, 300 case–parent trios have a power of 0.62 to detect a main genetic effect at a 0.05 significance level. Some genes in our study may be involved in human NTDs but cannot be detected with our sample size. In addition, typing one nonsynonymous SNP in a gene cannot capture the complete genetic diversity. For key genes more thorough interrogation requires exonic, intronic, and regulatory SNPs. The HapMap provides tagging SNPs based on the LD structure of the genomic region (Altshuler et al. 2005). Genes such as *MTR* are particularly problematic because high LD across a large region will make it very difficult to identify causative SNPs. Methionine cycle genes, such as *S*-adenosylhomocysteine hydrolase (*AHCY*; GenBank accession no. NM_000687), regulate the production of homocysteine and should also be investigated.

All SNPs were tested for HWE before analysis, primarily to identify genotyping errors. Of all the SNPs tested, only *MTHFD1* rs2236225 was out of HWE (*p* = 0.004) only in affected individuals. Departure from HWE in this study could result from genotyping error, selection, small sample size, or nonrandom mating. Unaffected individuals were in HWE for this SNP, potentially indicating an association, but no subsequent association was detected for this SNP. No other SNPs deviated from HWE, so there does not appear to be a widespread problem with the ascertainment of this sample set. Although this HWE deviation is interesting, it does not affect the overall outcome of the study because *MTHFD1* was not an implicated gene.

NTDs are a complex disorder involving many genetic and environmental factors. Future studies aimed at identifying these risk factors must approach the problem with a wide perspective including several genes and collecting as much environmental data as possible. Despite substantial efforts to associate NTDs with folate genes, there is no convincing evidence of an association for most of these genes. The role of folate in the etiology of NTDs could result from epigenetic effects or interactions with nonfolate genes. All previous research supports the multifactorial nature of NTDs underlining the necessity of multiple approaches in order to disentangle the contributors to this complex disorder.

## Figures and Tables

**Figure 1:**
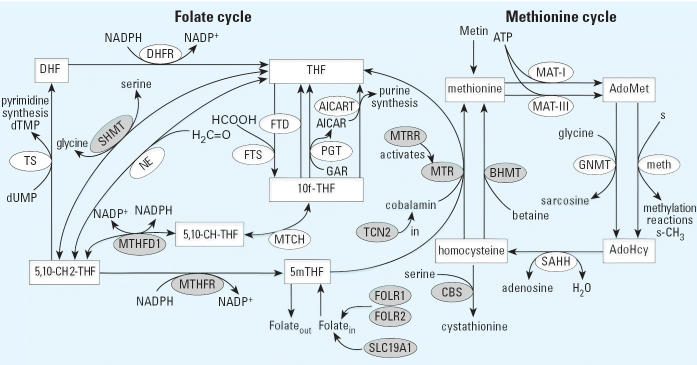
The folate and methionine cycles highlighting the 11 genes included in this study. Substrates are shown in rectangular boxes; enzymes are shown in ellipses. Adapted from Nijhout et al. (2004) and Reed et al. (2004). Substrate abbreviations: AdoHcy, *S*-adenosylhomocysteine; AdoMet, *S*-adenosylmethionine; DHF, dihydrofolate; 5,10-CH-THF, 5,10-methenyltetrahydrofolate; 5,10-CH2-THF, 5,10-methylenetetrahydrofo-late; THF, tetrahydrofolate; 5mTHF, 5-methyltetrahydrofolate; 10f-THF, 10-formyltetrahydrofolate. Enzyme abbreviations not included elsewhere: AICART, aminoimidazolecarboxamide ribotide transformylase; DHFR, dihydrofolate reductase; FTD, 10-formyltetrahydrofolate dehydrogenase; FTS, 10-formyltetrahydrofolate synthase; GNMT, glycine *N*-methyltransferase; MAT, methionine adenosyltransferase; meth, *S*-adenosylmethionine-dependent methyltransferases; MTCH, 5,10-methylenetetrahydrofolate cyclohydro-lase; NE, nonenzymatic interconversion of THF and 5,10-CH2-THF; PGT, phosphoribosyl glycinamidetrans-formylase; SAHH, *S*-adenosylhomocysteine hydrolase; TS, thymidylate synthase.

**Table 1:** Sample set details for the narrow (lumbo-sacral myelomeningocele only) and broad (any level NTD) diagnostic groups divided by maternal vitamin supplementation that was available for approximately 75% of mothers of affecteds.

Data set	Narrow	Broad
Full data set		
Families	279	304
Affecteds	297	332
Samples	1,158	1,259
Folate before conception		
Families	69	76
Affecteds	75	85
Samples	307	330
No folate before conception		
Families	141	149
Affecteds	151	165
Samples	617	653

**Table 2:** SNPs genotyped in the data set.

Gene symbol	Gene name	GenBank accession no.	rs no.	Type of SNP
*FOLR1*	folate receptor 1	NM_016725	rs2071010	Intronic
*FOLR2*	folate receptor 2	NM_000803	rs2298444	Intronic
*SLC19A1*	solute carrier family 19 member 1	U15939	rs1051266	Nonsynonymous
*TCN2*	transcobalamin II	NM_000355	rs1801198	Nonsynonymous
*MTHFD1*	methylenetetrahydrofolate dehydrogenase 1	J04031	rs2236225	Nonsynonymous
*SHMT1*	serine hydroxyl-methyltranferase 1	NM_004169	rs1979277	Nonsynonymous
*MTHFR*	5,10 methylene-tetrahydrofolate reductase	NM_005957	rs1801133	Nonsynonymous
			rs1801131	Nonsynonymous
*MTR*	5-methyltetrahydrofolate-homocysteine methyltransferase	NM_000254	rs10925235	Intronic
			rs12060570	Intronic
			rs10925250	Intronic
			rs1805087	Nonsynonymous
			rs4659743	intronic
*MTRR*	5-methyltetrahydrofolate-homocysteine methyltransferase reductase	AF025794	rs1801394	Nonsynonymous
*BHMT*	betaine-homocysteine methyltransferase	BC012616	rs651852	Intronic
			rs7700970	Intronic
			rs3733890	Nonsynonymous
			rs558133	Intronic
*CBS*	cystathionine-beta-synthase	NM_000071	rs234783	Intergenic
			rs234715	Intronic
			rs2851391	Intronic
			844ins68	—a
			rs1789953	Intronic
			rs4920037	Intronic
			rs1801181	Synonymous
			rs9325622	Intronic
			rs12613	Intronic
			rs412810	Intergenic

Gene annotations are from GenBank (http://www.ncbi.nih.gov/GenBank).

a 844ins68 is a 68-bp insertion in exon 8 of *CBS*.

**Table 3:** Single-gene *p*-values from significant association tests with an environmental stratum.

		Narrow		Broad	
Gene symbol	SNP data set	PDT	APL	PDT	APL
*BHMT*	rs3733890				
	Full data set	0.023*	0.058	0.025*	0.035*
	No suppl.	0.357	0.635	0.245	0.390
	Yes suppl.	0.027*	0.055	0.016*	0.027*
*BHMT*	rs558133				
	Full data set	0.114	0.124	0.026*	0.061
	No suppl.	0.765	0.983	0.296	0.657
	Yes suppl.	0.036*	0.139	0.012*	0.097
*MTHFR*	rs1801133				
	Full data set	0.203	0.112	0.317	0.263
	No suppl.	0.153	0.046*	0.235	0.102
	Yes suppl.	0.529	0.910	0.906	0.657
*MTR*	rs10925235				
	Full data set	0.877	0.794	0.715	0.865
	No suppl.	0.066	0.031*	0.040*	0.027*
	Yes suppl.	0.456	0.444	0.789	0.686
*MTR*	rs4659743				
	Full data set	0.885	0.426	0.547	0.375
	No suppl.	0.104	0.013*	0.041*	0.010*
	Yes suppl.	0.891	0.972	0.553	0.741
*CBS*	rs234715				
	Full data set	0.287	0.617	0.160	0.328
	No suppl.	0.056	0.190	0.015*	0.064
	Yes suppl.	0.527	0.562	0.435	0.683
*CBS*	rs4920037				
	Full data set	0.514	0.787	0.277	0.525
	No suppl.	0.122	0.213	0.037*	0.085
	Yes suppl.	0.423	0.509	0.435	0.650

Suppl., supplementation with folic acid before conception.

* *p* < 0.05.

**Table 4:** Linkage disequilibrium (*D*′ and *r*^2^) between SNPs in genes where more than three SNPs were genotyped in affected individuals.

*MTR*	rs10925235	rs12060570	rs10925250	rs1805087	rs4659743
rs10925235		0.966*	0.9	0.953*	0.973*
rs12060570	0.379		0.962*	0.961*	0.949*
rs10925250	0.122	0.176	1*	0.91*
rs1805087	0.131	0.169	0.976*		0.958*
rs4659743	0.946*	0.36	0.127	0.137	

*BHMT*	rs651852	rs7700970	rs3733890	rs558133
rs651852		0.884	0.632	0.226
rs7700970	0.316		0.367	0.334
rs3733890	0.14	0.124		1*
rs558133	0.028	0.02	0.162	

*CBS*	rs234783	rs234715	rs2851391	844ins68	rs1789953	rs4920037	rs1801181	rs9325622	rs12613	rs412810
rs234783		0.708	0.786	0.019	0.868	0.69	0.86	0.841	0.111	0.524
rs234715	0.192		0.963*	1*	1*	1*	1*	1*	1*	0.791
rs2851391	0.579	0.383		1*	0.915*	0.964*	0.985*	0.986*	1*	0.781
844ins68	0	0.028	0.068		1*	1*	0.897	0.789	0.971*	0.727
rs1789953	0.13	0.048	0.152	0.012		1*	1*	1*	0.999*	0.694
rs4920037	0.183	0.98*	0.395	0.028	0.049		1*	1*	1*	0.802
rs1801181	0.413	0.21	0.487	0.045	0.096	0.213		0.975*	1*	0.79
rs9325622	0.392	0.214	0.494	0.037	0.094	0.214	0.934*		0.883	0.803
rs12613	0.001	0.026	0.064	0.846	0.012	0.026	0.055	0.043		0.681
rs412810	0.149	0.441	0.372	0.02	0.032	0.456	0.185	0.201	0.017	

*D’* values are given above the diagonal; *r* ^2^ values are given below the diagonal.

* Linkage disequilibrium > 0.9.

**Table 5:** Single-gene *p*-values from significant association tests when stratified by preferential transmission of BHMT rs3733890 alleles.

Gene	SNP	Narrow		Broad	
symbol	data set	PDT	APL	PDT	APL
*SHMT1*	rs1979277				
	G allele	0.157	0.042*	0.066	0.020*
	A allele	0.096	0.204	0.101	0.247
	Neither	0.448	0.522	0.463	0.699
*MTR*	rs4659743				
	G allele	0.185	0.049*	0.052	0.015*
	A allele	0.691	0.184	0.701	0.193
	Neither	0.134	0.146	0.169	0.104
*CBS*	844ins68				
	G allele	0.033*	0.222	0.033*	0.217
	A allele	0.248	0.287	0.285	0.473
	Neither	0.842	0.318	1.000	0.560
*MTHFD1*	rs2236225				
	G allele	0.701	0.407	0.392	0.225
	A allele	0.064	0.214	0.016*	0.093
	Neither	0.822	0.666	0.915	0.739

* *p* < 0.05.

**Table 6:** Single-gene *p*-values from significant association tests when stratified by preferential transmission of MTHFR rs1801133 alleles.

Gene	SNP	Narrow		Broad	
symbol	data set	PDT	APL	PDT	APL
*BHMT*	rs3733890				
	C allele	0.647	0.815	0.405	0.991
	T allele	0.007*	0.027*	0.010*	0.047*
	Neither	0.335	0.171	0.463	0.143
*TCN2*	rs1801198				
	C allele	1.000	0.661	0.814	0.507
	T allele	0.056	0.092	0.045*	0.073
	Neither	0.829	0.959	1.000	0.932
*MTRR*	rs1801394				
	C allele	0.109	0.050	0.099	0.048*
	T allele	0.439	0.465	0.904	0.805
	Neither	0.473	0.601	0.502	0.717
*SHMT1*	rs1979277				
	C allele	0.317	0.370	0.279	0.318
	T allele	0.475	0.547	0.249	0.295
	Neither	0.050*	0.193	0.037*	0.190

* *p*< 0.05.

## References

[b1-ehp0114-001547] Abecasis GR, Cookson WO (2000). GOLD—graphical overview of linkage disequilibrium. BioInformatics.

[b2-ehp0114-001547] Afman LA, Lievers KJA, Kluijtmans LAJ, Trijbels FJM, Blom HJ (2003). Gene-gene interaction between the cystathionine beta-synthase 31 base pair variable number of tandem repeats and the methylenetetrahydrofolate reductase 677C→T polymorphism on homocysteine levels and risk for neural tube defects. Mol Genet Metab.

[b3-ehp0114-001547] Altshuler D, Brooks LD, Chakravarti A, Collins FS, Daly MJ, Donnelly P (2005). A haplotype map of the human genome. Nature.

[b4-ehp0114-001547] Belousov YS, Welch RA, Sanders S, Mills A, Kulchenko A, Dempcy R (2004). Single nucleotide polymorphism genotyping by two colour melting curve analysis using the MGB Eclipse Probe System in challenging sequence environment. Hum Genomics.

[b5-ehp0114-001547] De Marco P, Calevo MG, Moroni A, Arata L, Merello E, Finnell RH (2002). Study of MTHFR and MS polymorphisms as risk factors for NTD in the Italian population. J Hum Genet.

[b6-ehp0114-001547] Elwood JM Little J Elwood JH 1992. Epidemiology and Control of Neural Tube Defects. Oxford, UK:Oxford University Press.

[b7-ehp0114-001547] Essien FB, Wannberg SL (1993). Methionine but not folinic acid or vitamin-B12 alters the frequency of neural-tube defects in Axd mutant mice. J Nutr.

[b8-ehp0114-001547] Finkelstein JD, Martin JJ (1984). Methionine metabolism in mammals. Distribution of homocysteine between competing pathways. J Biol Chem.

[b9-ehp0114-001547] Frosst P, Blom HJ, Milos R, Goyette P, Sheppard CA, Matthews RG (1995). A candidate genetic risk factor for vascular disease: a common mutation in methylenetetrahydrofolate reductase. Nat Genet.

[b10-ehp0114-001547] Gonzalez-Herrera L, Garcia-Escalante G, Castillo-Zapata I, Canto-Herrera J, Ceballos-Quintal J, Pinto-Escalante D (2002). Frequency of the thermolabile variant C677T in the MTHFR gene and lack of association with neural tube defects in the State of Yucatan, Mexico. Clin Genet.

[b11-ehp0114-001547] Gueant-Rodriguez RM, Rendeli C, Namour B, Venuti L, Romano A, Anello G (2003). Transcobalamin and methionine synthase reductase mutated polymorphisms aggravate the risk of neural tube defects in humans. Neurosci Let.

[b12-ehp0114-001547] International HapMap Project (2005). A haplotype map of the human genome. Nature.

[b13-ehp0114-001547] Kang SS (1996). Treatment of hyperhomocyst(e)inemia: physiological basis. J Nutr.

[b14-ehp0114-001547] Martin ER, Bass MP, Hauser ER, Kaplan NL (2003). Accounting for linkage in family-based tests of association with missing parental genotypes. Am J Hum Genet.

[b15-ehp0114-001547] Martin ER, Monks SA, Warren LL, Kaplan NL (2000). A test for linkage and association in general pedigrees: the pedigree disequilibrium test. Am J Hum Genet.

[b16-ehp0114-001547] Mills JL, McPartlin JM, Kirke PN, Lee YJ, Conley MR, Weir DG (1995). Homocysteine metabolism in pregnancies complicated by neural-tube defects. Lancet.

[b17-ehp0114-001547] Morin I, Platt R, Weisberg I, Sabbaghian N, Wu Q, Garrow TA (2003). Common variant in betaine-homocysteine methyl-transferase (BHMT) and risk for spina bifida. Am J Med Genet.

[b18-ehp0114-001547] Morrison K, Papapetrou C, Hol FA, Mariman EC, Lynch SA, Burn J (1998). Susceptibility to spina bifida; an association study of five candidate genes. Ann Hum Genet.

[b19-ehp0114-001547] MRC Vitamin Study Research Group (1991). Prevention of neural tube defects: results of the Medical Research Council Vitamin Study. Lancet.

[b20-ehp0114-001547] Nijhout HF, Reed MC, Budu P, Ulrich CM (2004). A mathematical model of the folate cycle: new insights into folate homeo-stasis. J Biol Chem.

[b21-ehp0114-001547] Posey DL, Khoury MJ, Mulinare J, Adams MJ, Ou CY (1996). Is mutated MTHFR a risk factor for neural tube defects?. Lancet.

[b22-ehp0114-001547] Ramsbottom D, Scott JM, Molloy A, Weir DG, Kirke PN, Mills JL (1997). Are common mutations of cystathionine beta-synthase involved in the aetiology of neural tube defects?. Clin Genet.

[b23-ehp0114-001547] Razin A, Kantor B (2005). DNA methylation in epigenetic control of gene expression. Prog Mol Subcell Biol.

[b24-ehp0114-001547] Reed MC, Nijhout HF, Sparks R, Ulrich CM (2004). A mathematical model of the methionine cycle. J Theor Biol.

[b25-ehp0114-001547] Revilla JIG, Hernandez FN, Martin MTC, Salvador MT, Romero JG (2003). C677T and A1298C MTHFR polymorphisms in the etiology of neural tube defects in Spanish population. Med Clin.

[b26-ehp0114-001547] Rosenquist TH, Finnell RH (2001). Genes, folate and homocysteine in embryonic development. Proc Nutr Soc.

[b27-ehp0114-001547] Rosenquist TH, Ratashak SA, Selhub J (1996). Homocysteine induces congenital defects of the heart and neural tube: effect of folic acid. Proc Natl Acad Sci USA.

[b28-ehp0114-001547] Shields DC, Kirke PN, Mills JL, Ramsbottom D, Molloy AM, Burke H (1999). The “thermolabile” variant of methylene-tetrahydrofolate reductase and neural tube defects: an evaluation of the genetic risk and the relative importance of the genotypes of the embryo and the mother. Am J Hum Genet.

[b29-ehp0114-001547] Slesinski MJ, Subar AF, Kahle LL (1996). Dietary intake of fat, fiber and other nutrients is related to the use of vitamin and mineral supplements in the United States: the 1992 National Health Interview Survey. J Nutr.

[b30-ehp0114-001547] Speer MC, Nye J, McLone D, Worley G, Melvin EC, Viles KD (1999). Possible interaction of genotypes at cystathionine beta-synthase and methylenetetrahydrofolate reductase (MTHFR) in neural tube defects. NTD Collaborative Group. Clin Genet.

[b31-ehp0114-001547] Steegers-Theunissen RPM, Boers GHJ, Trijbels FJM, Finkelstein JD, Blom HJ, Thomas CMG (1994). Maternal hyperhomo-cysteinemia—a risk factor for neural-tube defects. Metab Clin Exp.

[b32-ehp0114-001547] Stegmann K, Ziegler A, Ngo ETKM, Kohlschmidt N, Schröter B, Ermert A (1999). Linkage disequilibrium of MTHFR genotypes 677C/T-1298A/C in the German population and association studies in probands with neural tube defects (NTD). Am J Med Genet.

[b33-ehp0114-001547] Trembath D, Sherbondy AL, Vandyke DC, Shaw GM, Todoroff K, Lammer EJ (1999). Analysis of select folate pathway genes, PAX3, and human T in a midwestern neural tube defect population. Teratology.

[b34-ehp0114-001547] van der Put NM, Steegers-Theunissen RP, Frosst P, Trijbels FJ, Eskes TK, van den Heuvel LP (1995). Mutated methyl-enetetrahydrofolate reductase as a risk factor for spina bifida. Lancet.

[b35-ehp0114-001547] van der Put NM, Thomas CMG, Eskes TK, Trijbels FJ, Steegers-Theunissen RP, Mariman EC (1997). Altered folate and vitamin B-12 metabolism in families with spina bifida offspring. QJM.

[b36-ehp0114-001547] van der Put NM, van den Heuvel LP, Steegers-Theunissen RP, Trijbels FJ, Eskes TK, Mariman EC (1996). Decreased methylene tetrahydrofolate reductase activity due to the 677C → T mutation in families with spina bifida offspring. J Mol Med.

[b37-ehp0114-001547] Waterland RA, Jirtle RL (2003). Transposable elements: targets for early nutritional effects on epigenetic gene regulation. Mol Cell Biol.

[b38-ehp0114-001547] Weir BS 1996. Genetic Data Analysis II: Methods for Discrete Population Genetic Data. Sunderland, MA:Sinaur Associates

[b39-ehp0114-001547] Weisberg IS, Park E, Ballman KV, Berger P, Nunn M, Suh DS (2003). Investigations of a common genetic methyltransferase (BHMT) variant in betaine-homocysteine in coronary artery disease. Atherosclerosis.

[b40-ehp0114-001547] Wilson A, Platt R, Wu Q, Leclerc D, Christensen B, Yang H (1999). A common variant in methionine synthase reductase combined with low cobalamin (vitamin B-12) increases risk for spina bifida. Mol Genet Metab.

[b41-ehp0114-001547] Zhu HP, Curry S, Wen S, Wicker NJ, Shaw GM, Lammer EJ (2005). Are the betaine-homocysteine methyltransferase (*BHMT* and *BHMT2*) genes risk factors for spina bifida and orofacial clefts?. Am J Med Genet.

